# Polyethylene Terephthalate Composite Films with Enhanced Flame Retardancy and Gas Barrier Properties via Self-Assembly Nanocoating

**DOI:** 10.3390/nano13132018

**Published:** 2023-07-06

**Authors:** Tao Zou, Lei Kang, Dongqiao Zhang, Jieyi Li, Zefeng Zheng, Xiaohong Peng

**Affiliations:** 1 School of Materials Science and Engineering, South China University of Technology, Guangzhou 510640, China; 2 School of Civil Engineering, Guangzhou University, Guangzhou 510006, China; 3 Shaanxi Engineering Laboratory of High Performance Concrete, Shaanxi Railway Institute, Weinan 714099, China; 4 School of Chemistry and Chemical Engineering, Guangzhou University, Guangzhou 510006, China; 5 Key Laboratory of Polymer Processing Engineering (South China University of Technology), Ministry of Education, Guangzhou 510640, China

**Keywords:** self-assembly nanocoating, composite film, flame retardancy, gas barrier property

## Abstract

The flammability and gas barrier properties are essential for package material. Herein, a highly-oriented self-assembly nanocoating composed of polyvinyl alcohol (PVA) and montmorillonite (MMT) was prepared for endowing polyethylene terephthalate (PET) films with excellent flame retardancy and gas barrier properties. The specific regular nanosheet structure of the PVA/MMT composite nanocoating was confirmed by Fourier transform infrared (FTIR) and X-ray diffraction (XRD). Thermogravimetric analysis (TGA) and the vertical burning test (VBT) suggested that the thermal stability and flame-retardancy of the coated PET films were considerably improved with more pick-up of the resulting nanocoating. When reaching 650 °C, there was still 22.6% char residual left for coated PET film, while only 6% char residual left for pristine PET film. During the vertical burning test, the flame did not spread through the whole PET film with the protection of PVA/MMT nanocoating, and no afterflame was observed. Scanning electron microscopy (SEM) is consistent with vertical burning test, proving that the thermal stability and flame retardancy of coated PET films were considerably enhanced with the increment of PVA/MMT. Thanks to the multi-layer structure, PVA/MMT nanocoating could effectively improve the gas barrier properties of PET films, and the oxygen vapor transmittance rate and water vapor transmittance rate of PET films were more than four hundred times lower and 30% lower than those of neat PET film. Our work demonstrates that bi-functional flame retardant and gas barrier materials could be gained via constructing inorganic/organic highly-oriented self-assembly nanocoating, which is promising in the area of packaging.

## 1. Introduction

Packaging materials have evolved from natural materials (wood, glass and metal, etc.) to synergistic polymer (plastic and synergistic fibers, etc.). As a conventional packaging material, plastic is popular for the weight/cost advantage, flexibility, and portability. However, as a traditional plastic package material, polyethylene terephthalate (PET) is intrinsically flammable [1]. Once a fire accident happens, plastic will accelerate the fire and flame spread, and it might probably cause death and property loss. In 2021, US Local fire departments responded to 1,353,500 fires. These fires caused 3800 civilian deaths, 14,700 civilian injuries, and $15.9 billion in property damage [2]. Meanwhile, the gas barrier property of PET is still limited as a packaging material, but low transfer of oxygen and moisture is required to extend the shelf-life in a packaging area [3,4,5]. Hence, the improvement of the flame retardancy and barrier performance for PET-based packaging material is significant.

The layer-by-layer (LBL) self-assembly technique is very effective in providing flame retardancy and gas barrier characteristics to different substrates by depositing various polyelectrolytes or particles via hydrogen bonding, covalent bonding, or electrostatic force [6]. Since 2009, Grunlan et al. have been the pioneer in LBL, inspiring many researchers to explore LBL in the development of flame retardancy and barrier functional materials [7,8,9,10]. Laufer et al. applied LBL technique to endow thin polylactic acid (PLA) films with exceptional flame retardant and oxygen barrier properties. A 30-bilayer (CH pH 6-MMT) nanocoating reduces the oxygen permeability of polylactic acid film by four orders of magnitude. The coating system completely stops the melting of a flexible polyurethane foam with just 10 bilayers [7]. Laachachi et al. [11]. created highly efficient intumescent flame-retardant coatings with the diffusion of polyphosphates (PSPs) in exponentially growing “layer-by-layer” montmorillonite (MMT)–poly(allylamine) (PAH) films, which were applied to improve the flame retardancy and the oxygen property of PLA. The OTR values show that (PAH-MMT)_n_ films have a very low oxygen permeability, even though the measurements were performed in the presence of 90% relative humidity. The presence of (PAH-MMT)_n_ coatings at the surface of PLA significantly increases the time to ignition (TTI) and decreases the maximum of HRR in comparison to uncoated PLA. The LBL technique has the advantage of controlling the thickness, transparency, flame retardancy, and gas barrier properties by adjusting the layers in a delicate way. However, the LBL technique is labor-intensive for multiple cycles of immersion and rise processes, which are hard in relation to a large-scale [12].

Inorganic/organic composite-layered structures possess an excellent barrier property and flame retardancy. Therefore, many researchers tried to construct an inorganic/organic composite-layered structure onto different substrates to gain satisfactory flame retardant and barrier performance [13,14]. Ding et al. [15] constructed hybrid nanocoating with nacre-like microstructure via a facile one-step co-assembly process, demonstrating exceptional flame retardancy, high transparency, and superior mechanical and barrier characteristics in different substrates with regular or irregular surfaces, which provided a very effective path to gain biomimetic inorganic/organic composite materials with multi-functionalities. Guin et al. [16] incorporated amine salts into nanoclay to achieve nanoplatelets in stack. The thick clay-based thin films endowed polystyrene plate exceptional flame resistance, as well as oxygen transmission rates below 0.009 cc m^−2^ d^−1^ atm^−1^. Meng et al. [12] fabricated an organic/inorganic hybrid sub-micron coating with two kinds of clay and PVA for depositing onto the surface of PET film via facile co-assembly. The peak of heat release rate and total heat release of coated PET samples were reduced by 67.4% and 45.3%, respectively, and the oxygen transmission rate was reduced to almost zero at less than five layers of coating. Liu et al. [17] prepared nanopaper with a multilayered structure via nanofibrillated cellulose (NFC) and MMT. The hybrid nanopaper shows a storage modulus of 11.0 GPa at room temperature, and it could immediately self-extinguish upon removal of the flame, even at 50 wt.% MMT content. The OTR of the NFC–MTM hybrid nanopaper was much lower than that of pure NFC. The layered structure of the hybrid nanopaper and the in-plane orientation of MMT increase the diffusion path length of small gas molecules.

Herein, a highly-oriented self-assembly nanocoating composed of polyvinyl alcohol (PVA) and montmorillonite (MMT) via flow-induced orientation was prepared to endow polyethylene terephthalate (PET) films with flame retardancy and gas barrier properties. The chemical and specific regular nanosheet structure of the PVA/MMT composite nanocoating was studied by Fourier transform infrared (FTIR) and X-ray diffraction (XRD). The morphology of coated PET films before and after the vertical burning test was explored by scanning electron microscopy (SEM). Herein, we mainly focus on the impact of coating times on the thermostability, flame retardancy, and gas barrier properties (including oxygen and water vapor) of the coated PET films, and we tried to explore the inner course.

## 2. Materials and Methods

### 2.1. Materials

Ethanol anhydrous (EtOH, ≥99.7%) was supplied by Tianjin Zhiyuan Chemical Reagent Co., Ltd. (Tianjin, China). Deionized (DI) water and hydrochloric acid (HCl, 37 wt%) were purchased from Guangzhou Chemical Reagent Factory (Guangzhou, China). Sodium montmorillonite (MMT) and polyvinyl alcohol (PVA) were provided by Minerals Technologies Inc. (New York, NY, USA) and Kuraray Co., Ltd. (Tokyo, Japan), respectively. Polyethylene terephthalate (PET) film and glutaraldehyde (GA) were obtained from Hongmei New Material Co., Ltd. (Shenzhen, China) and BASF chemicals company limited (Hamburg, Germany).

### 2.2. Methods

#### 2.2.1. Preparation of PVA/MMT Composite Nanocoating

Firstly, MMT was mixed with DI water, which was stirred overnight at room temperature. PVA was added to the well dispersed MMT solution, which was stirred for 4 h, and the solid content was 1.5 wt.% with a MMT/PVA weight ratio as 1/1. After that, GA was introduced in the solution above, with stirring for 15 min, and the weight ratio of GA/PVA/MMT was 1/5/5, and they were ultrasonicated for 0.5 h to obtain a well dispersed PVA/MMT mixture. Eventually, a self-assembly co-cross-linked PVA/MMT nanocoating was prepared.

#### 2.2.2. Fabrication of PVA/MMT-Coated PET Films

Before dip-coating, PET films were washed with DI water and EtOH alternately, and they were dried at 60 °C for 30 min. Then, these dried PET films were dip-coated with the obtained PVA/MMT composite nanocoating 2, 4, and 6 times, respectively. After being dried at 60 °C for 20 min, PVA/MMT-coated PET films were prepared, and they were noted as C-2, C-4, and C-6 according to the coating times, respectively, as shown in Table 1. It should be noted that, after the pristine PET film was coated the first time and was put into an oven to become dried, we switched the sample in a vertical direction after every drying, and we carried out the next dipping and subsequent drying to minimize non-uniformity.

### 2.3. Characterization

X-ray diffraction (XRD) patterns of the samples were recorded on a Bruker D5005 diffractometer (Bruker, Zurich, Switzerland) using a graphite monochromator with Cu Ka radiation (k = 1.5406 Å). The scanning speed was 2 deg/min with a scanning angle ranging from 5 to 40°. The testing current and voltage were 40 mA and 40 kV, respectively. MMT was fully filled into a circular plastic holder, while the PVA/MMT coating was coated onto a monocrystalline silicon piece and was dried at 80 °C for 40 min, which was transferred into a circular plastic holder then. The scanning electron microscopy (SEM) test was conducted to study the morphology and microstructure of all samples before and after the vertical burning test by a Quanta200 (FEI, Lexington, KY, USA) at an accelerating voltage of 10 kV. The samples were adhered to a metal with the connection of a conductive tape, and this was followed by a sputter coating with a thin layer of Au/Pd prior to SEM imaging. Attenuated total reflectance (ATR) Fourier transform infrared (FTIR) spectra were collected at room temperature in the range of 400–4000 cm^−1^ on a Nicolet Magna 560 FTIR spectrophotometer equipped with a diamond crystal at a resolution of 1 cm^−1^ after 32 scans. The MMT solution was dipped into a KBr sample, while PVA/MMT coating was coated onto a monocrystalline silicon piece and was dried at 80 °C for 40 min. A thermogravimetric analyzer was used to study the thermal stability of coated PET films by a TG209 F3 (NETZSCH, Selb, Germany) at a heat rate of 20 °C/min under an air atmosphere with a flow of 40 mL/min. All samples were scanned from room temperature to 650 °C. Vertical burning tests (VBT) were carried out in a flame cabinet according to ASTM D6413-08. The control and coated PET films with a size of 2 inch × 6 inch were exposed to a direct flame from a Bunsen burner. Removing the Bunsen burner after 12 s, the damaged lengths and afterflame time were recorded. The barrier properties of the control and coated PET films were studied by oxygen and water vapor barrier tests. The oxygen barrier test was conducted on Oxtran 2/21 ML (MOCON, Brooklyn Park, MN, USA) at 23 °C and 0% RH, following ASTM D3985. The water vapor barrier test was carried out by using Permatran-W 3/33 (MOCON, USA) at 23 °C and 100% RH, following ASTM F-1249.

## 3. Results and Discussion

### 3.1. Design Strategy and Self-Assembly Mechanism

**Figure 1:**
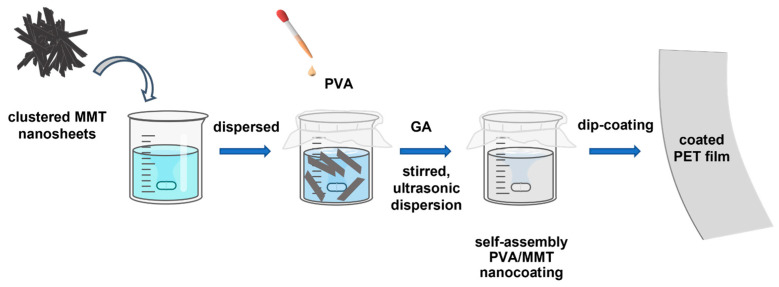
A schematic representation of the preparation of the self-assembled PVA/MMT nanocoating and coated PET film.

The self-assembly of the PVA/MMT nanocoating is illustrated in Figure 1. Under mechanical stirring, MMT was firstly dispersed in water with the help of a hydrogen bond. Thanks to the hydrogen bond interaction between MMT and PVA, MMT nanosheets were exfoliated by PVA, forming self-assembly PVA/MMT composite nanosheets. When the PET film coated with PVA/MMT was vertically suspended, this generated flow-induced orientation among PVA and MMT, which could be due to gravity. Consequently, this flow-induced orientation contributed to the formation of a dense self-assembly PVA/MMT nanocoating.

### 3.2. Composition and Structure

**Figure 2:**
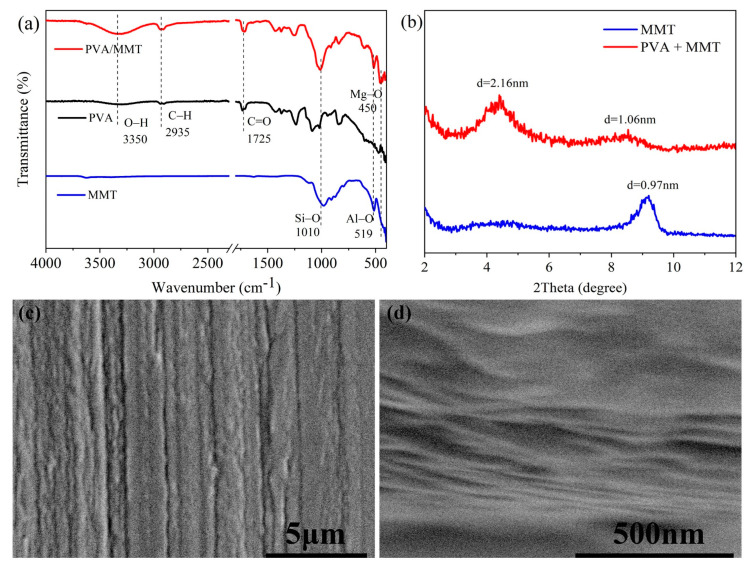
(**a**) The FTIR spectra of MMT, PVA, and PVA/MMT; (**b**) The XRD patterns of PVA/MMT and neat MMT; (**c**,**d**) The SEM images of PVA/MMT coated PET film in cross section direction.

The chemical structure of PVA/MMT nanocoating was investigated by FTIR. As shown in Figure 2a, the peaks present at 1010, 519 and 450 cm^−1^ are associated with the stretching vibration of Si-O, Al-O, and Mg-O from MMT, respectively [18,19]. The stretching vibration of O-H shows at ca. 3350 cm^−1^, contributing to hydroxyl groups in PVA [20]. Because the ester groups in PVA are partially hydrolyzed, the peak at ca. 1725 cm^−1^ corresponds to C=O [21]. The brand at 2935 cm^−1^ is due to the stretching vibration of C-H from PVA. For the spectrum of PVA/MMT, the peaks above all appeared.

XRD tests were carried out to characterize the self-assembly PVA/MMT composite nanosheet structure. As shown in Figure 2b, neat MMT shows a peak at 9.2°, corresponding to the basal diffraction of the stacked MMT nanosheets [22,23]. The diffraction peak of PVA/MMT arrives at 4.4°, and the interlayer distance of reassembled MMT nanosheets increased from 0.97 to 2.16 nm, which is related to the intercalation of PVA into MMT nanosheets [24]. The combination of FTIR and XRD has demonstrated that PVA/MMT nanocoating with a specific nanosheet structure has been successfully prepared.

SEM was used to investigate the nanosheet structure of PVA/MMT coating. As shown in Figure 2c,d, PVA/MMT coating appeared highly oriented. In the SEM test, metal nanoparticles were sputtered onto the cross-section of coated PET film to become conductive in this test, so the PVA/MMT nanosheet was filled with a metal nanoparticle. Restricted by the instrument accuracy, the picture in Figure 2d for showing the nanosheet structure of PVA was not clear.

### 3.3. Thermal Stability and Flame Retardancy

**Figure 3:**
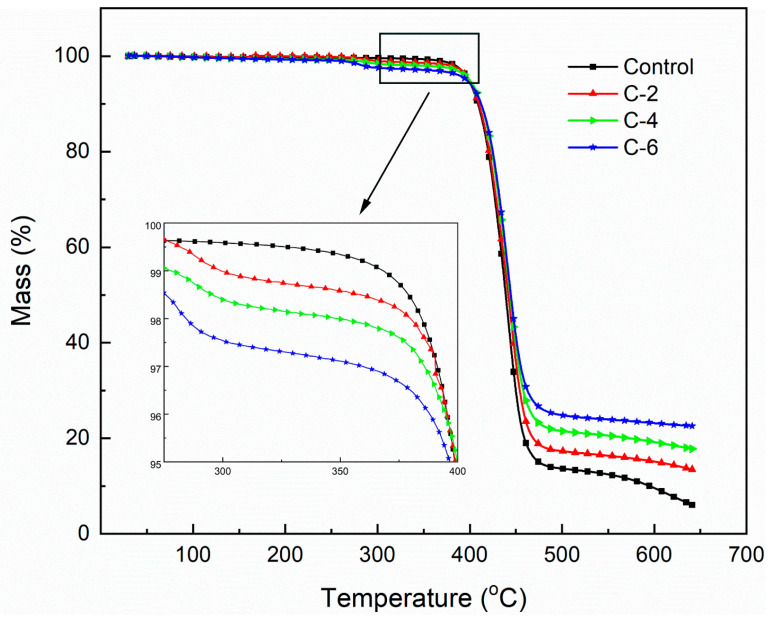
Thermostability of the control and coated PET films in the air.

TGA were conducted to study the thermal stability of neat and coated PET films. As shown in Figure 3, the main weight loss of neat PET film occurred from 375 °C to 470 °C, originating from the thermal degradation of PET main chain [25,26]. With the temperature rising, the PET chain fragments were further decomposed to volatile gases. All the coated PEF films displayed three stages of weight loss. Compared with the temperature of the initial weight loss for neat PET (at around 300 °C), the initial weight loss for all the coated PET films started earlier (at around 260 °C), which is related to the dehydration of PVA before the breakage of PET chains [27,28]. However, C-2, C-4, and C-6 still maintained 19.4%, 24.1%, and 27.5% at around 470 °C after drift weight loss, respectively, though early weight loss appeared in lower temperature. When reaching 650 °C, there were still 13.8%, 18.0%, and 22.6% char residuals for C-2, C-4, and C-6, respectively. Herein, MMT acted as a physical protective layer, and it was transformed to a dense intumescent heat-resistant layer combined with PVA when encountering fire [29]. Hence, PET was effectively protected by PVA/MMT nanocoating. According to the above analysis, the thermal stability of the PET substrate was considerably improved because PVA/MMT self-assembly nanocoating contributed to the formation of a thick and effective intumescent heat-resistant layer.

**Figure 4:**
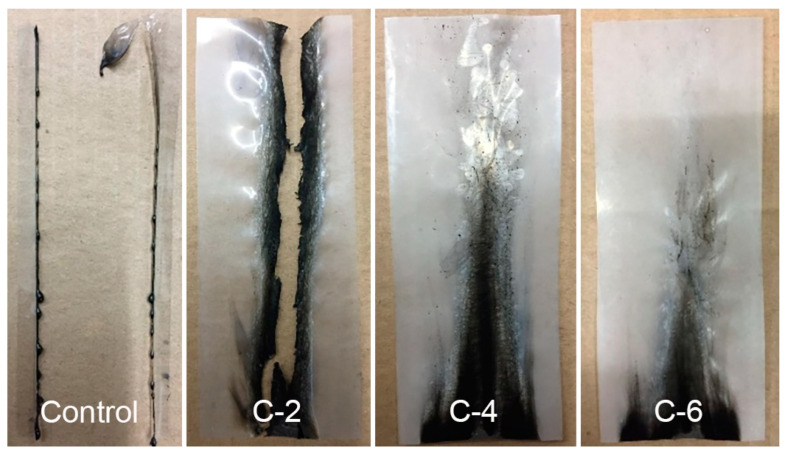
Pictures of the control and coated PET films after the vertical burning test.

The flame retardancy of PET-PVA/MMT films was studied by the vertical burning test. The PET film, with a size of 2 inches × 6 inches, was exposed to a direct flame from a Bunsen burner. Removing the Bunsen burner after 12 s, the damaged lengths and the afterflame were recorded. As shown in Figure 4, the control sample was ignited immediately and burned out eventually, indicating the extreme flammability of the PET film. For C-2, the flame spread through a large area of C-2, but the film was not fully damaged. Meanwhile, the combustion lengths of C-4 and C-6 were just 4.3 and 3 inches, respectively. Moreover, no afterflame was observed during the VBT tests of C-4 and C-6. Therefore, PVA/MMT nanocoating could effectively protect PET films from causing destruction by generating thermal char as a protective shield.

In summary, compared with the pure PET film, all coated PET films were endowed with flame retardancy and anti-dropping properties. Benefiting from the orientation of intercalated PVA into MMT nanosheets under sonication, a dense flame-retardant char layer was formed by nanocoating during the VBT test, which inhibited the oxygen diffusion and heat transfer, contributing to the suppression of the growth of flame. Moreover, the flame resistance of the composite PET films could be improved by increasing the coating times and the pickup of PVA/MMT.

### 3.4. Morphology and Microstructure

**Figure 5:**
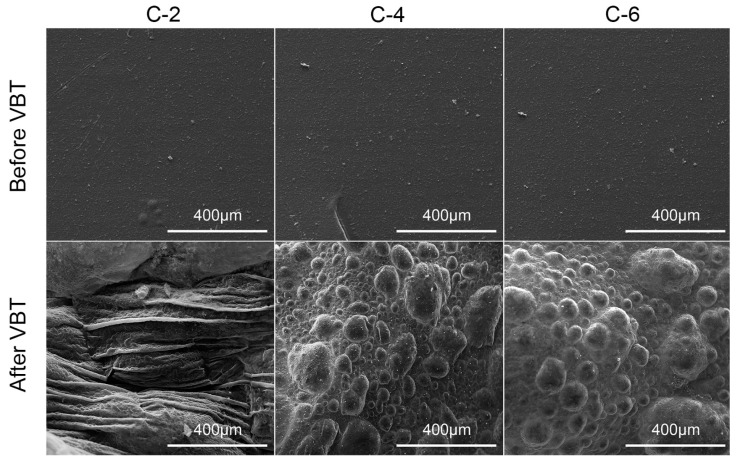
Low-magnification SEM images of the coated PET films before and after VBT.

**Figure 6:**
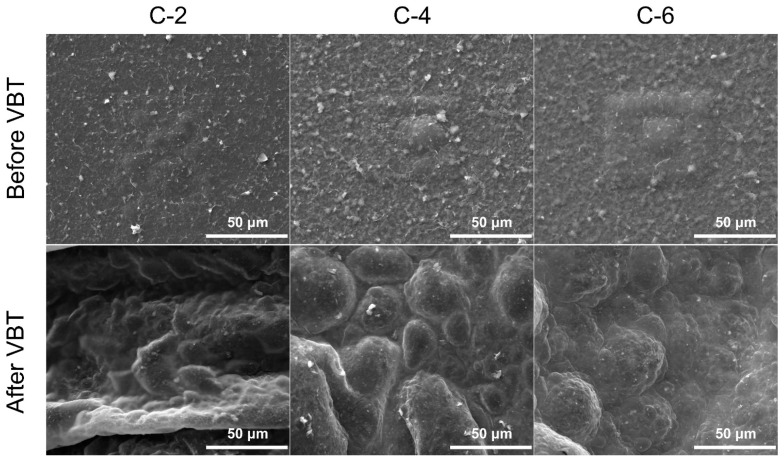
High-magnification SEM images of the coated PET films before and after VBT.

SEM was employed to investigate the morphology and the microstructure of coated PET films, as exhibited in Figure 5 and Figure 6. Before VBT, the surface of the coated samples was relatively coarse due to the interaction of PVA and MMT. After VBT, the surface of C-2 seemed to be filled with shrinking fibers, indicating that the PVA/MMT composite nanocoating was insufficient to protect the PET substrate. In contrast, a large amount of intumescent bubbles could be observed on the surface of C-4 and C-6 after VBT. These intense carbonaceous bubbles stemmed from the CO_2_ and other escape gases captured by the dense highly-orientated char layer produced during the combustion of PVA/MMT films. The intumescent char could effectively restrain the flame, heat, and oxygen from devastating the PET substrate. The SEM images are consistent with the vertical burning test, proving that the thermal stability and flame retardancy of coated PET films were considerably enhanced with the increment of PVA/MMT.

### 3.5. Gas Barrier Property

**Figure 7:**
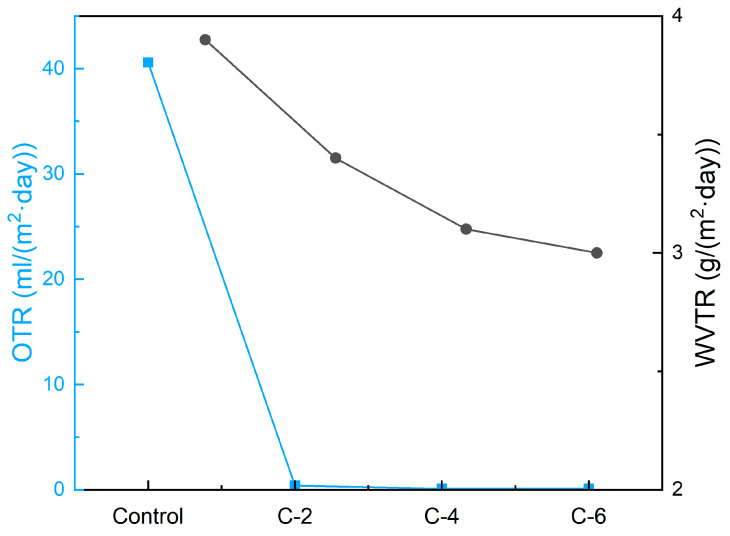
The barrier properties of coated PET films.

As shown in Figure 7, oxygen vapor transmittance rate (OTR) and water vapor transmittance rate (WVTR) were tested to characterize the gas barrier property of coated PET films. For C-2, the OTR was as low as 0.4 mL/(m^2^·day), which was 100 times less than that of neat PET films, indicating that the PVA/MMT nanocoating could effectively enhance the oxygen barrier property of PET film. It should also be noted that C-4 and C-6 showed lower OTR with regards to increasing the pick-up of PVA/MMT, which is almost four times less than that of C-2. The increased pickup of PVA/MMT highly-oriented nanocoating directly contributed to the formation of thicker dense composite nanosheets for endowing the PET films with better oxygen barrier property.

The WVTR of C-2, C-4, and C-6 were 3.4, 3.1, and 3.0 g/(m^2^·day), respectively, exhibiting a downtrend, and the WVTR of all coated films were lower than that of neat PET film. The improvement of water vapor barrier performance is not as evident as that of the oxygen barrier property. Though dense nanosheets constructed by self-assembly PVA/MMT could improve the gas barrier property of PET film, the abundant hydroxyl groups of PVA favor the absorption of water vapor. Consequently, the improvement of the water vapor barrier property for the coated films was inapparent. In summary, the OTR and WVTR were both improved because of the dense nanosheets composed of PVA and MMT.

## 4. Conclusions

A highly-oriented self-assembly nanocoating composed of PVA and MMT was successfully prepared for enhancing the flame retardancy and barrier properties of PET films. FTIR and XRD demonstrated that PVA/MMT nanocoating possesses a regular nanosheet structure with a specific sheet structure. When reaching 650 °C, there was still 22.6% char residual left for coated PET film, while only 6% char residual left for pristine PET film. During the vertical burning test, the flame did not spread through the whole PET film with the protection of PVA/MMT nanocoating, and no afterflame was observed. TGA and VBT tests have shown that the thermal stability and flame retardancy of PET films were remarkably enhanced with regards to the protection of the PVA/MMT nanocoating, which were further improved with more pick-up of PVA/MMT. The nanocoating would be transformed into a thermal bubble-like char to protect PET films when meeting high-temperature heat and fire. The SEM images are consistent with the vertical burning test, proving that the thermal stability and the flame retardancy of coated PET films were considerably enhanced with the increment of PVA/MMT. Thanks to the multi-layer structure, the PVA/MMT coating could effectively improve the gas barrier properties of PET films, and the oxygen vapor transmittance rate and water vapor transmittance rate of PET films were more than four hundred times lower and 30% lower than those of neat PET film. Our work demonstrates that bi-functional flame retardant and gas barrier PET films could be gained via the self-assembly of PVA and MMT, which is promising in the area of packaging.

## Figures and Tables

**Table 1:** Coating times and the thickness of the coated samples.

Sample ID	Coating Times	Coating Thickness (nm)
C-2	2	372 ± 36
C-4	4	645 ± 25
C-6	6	848 ± 22

## Data Availability

Data are available upon request from the authors.
